# The Successful Diagnosis and Typing of Systemic Amyloidosis Using A Microwave-Assisted Filter-Aided Fast Sample Preparation Method and LC/MS/MS Analysis

**DOI:** 10.1371/journal.pone.0127180

**Published:** 2015-05-18

**Authors:** Weiyi Sun, Jian Sun, Lili Zou, Kaini Shen, Dingrong Zhong, Daobin Zhou, Wei Sun, Jian Li

**Affiliations:** 1 Core Facility of Instrument, Institute of Basic Medical Sciences, Chinese Academy of Medical Sciences, School of Basic Medicine, Peking Union Medical College, Beijing, China; 2 Department of Hematology, Peking Union Medical College Hospital, Peking Union Medical College & Chinese Academy of Medical Sciences, Beijing, China; 3 Department of Pathology, Peking Union Medical College Hospital, Peking Union Medical College & Chinese Academy of Medical Sciences, Beijing, China; Kermanshah University of Medical Sciences, IRAN, ISLAMIC REPUBLIC OF

## Abstract

Laser microdissection followed by mass spectrometry has been successfully used for amyloid typing. However, sample contamination can interfere with proteomic analysis, and overnight digestion limits the analytical throughput. Moreover, current quantitative analysis methods are based on the spectrum count, which ignores differences in protein length and may lead to misdiagnoses. Here, we developed a microwave-assisted filter-aided sample preparation (maFASP) method that can efficiently remove contaminants with a 10-kDa cutoff ultrafiltration unit and can accelerate the digestion process with the assistance of a microwave. Additionally, two parameters (P- and D-scores) based on the exponentially modified protein abundance index were developed to define the existence of amyloid deposits and those causative proteins with the greatest abundance. Using our protocol, twenty cases of systemic amyloidosis that were well-typed according to clinical diagnostic standards (training group) and another twenty-four cases without subtype diagnoses (validation group) were analyzed. Using this approach, sample preparation could be completed within four hours. We successfully subtyped 100% of the cases in the training group, and the diagnostic success rate in the validation group was 91.7%. This maFASP-aided proteomic protocol represents an efficient approach for amyloid diagnosis and subtyping, particularly for serum-contaminated samples.

## Introduction

Amyloidoses are a class of diseases characterized by the extracellular deposition of misfolded proteins in an insoluble β-pleated conformation [1]. To date, more than 30 different proteins have been recognized as causative agents of human amyloidosis, 14 of which are associated with systemic forms [2]. Clinically, the most common amyloidogenic proteins are immunoglobulin light chains, serum amyloid A protein (SAA) and transthyretin (TTR) [3]. These various subtypes present overlapping clinical manifestations but differ in their pathogenesis, prognosis and treatment. Thus, precise amyloid typing is critical for effective disease management.

In routine practice, traditional antibody-based methods, such as immunohistochemistry and immunofluorescence, are widely used for amyloid typing. However, disadvantages that include limited antibody availability, contamination from serum proteins and loss of epitopes recognized by the antibody significantly impair the sensitivity and specificity of diagnoses [4, 5]. A review of previously published series has shown a wide range of success rates for immunohistochemical amyloid typing, ranging from 38% to 87%, whereas the success rate of immunofluorescence in frozen tissues, which are typically used in renal pathology, ranges between 65% and 87% [6].

Recently, a laser microdissection- and mass spectrometry (MS)-based proteomic approach (LMD/MS) has revolutionized the field of amyloid typing. In 2009, Vrana and colleagues established this method for amyloid typing in routine clinical formalin-fixed, paraffin-embedded (FFPE) specimens [7]. The authors successfully identified amyloid subtypes for fifty cases that were well-characterized by the gold standard with 100% sensitivity and specificity. For another forty-one cases of amyloidosis, the sensitivity of the LMD/MS typing method reached 98%. Subsequently, LMD/MS methods have been successfully applied for subtype diagnosis in renal amyloidosis and amyloid neuropathy [8–10]. These studies have demonstrated that the LMD/MS typing method is a useful tool for amyloid typing and show its superiority to conventional methods.

Although MS-based proteomics provides an alternative method for amyloid diagnosis, some pivotal issues should be further investigated. The first issue is contamination during sample preparation. Prior to liquid chromatography/tandem MS (LC/MS/MS) analysis, paraffin wax should be removed from FFPE specimens, and Congo red staining and laser microdissection are then used to locate and isolate amyloid areas. During these processes, contaminants are easily introduced, which can interfere with MS analyses. A second issue is protein digestion. Although conventional overnight trypsin digestion has been widely used in previous studies, it is time-consuming and low-throughput. Because protein digestion is the rate-limiting step prior to LC/MS/MS analysis, speeding up this step will facilitate the more rapid clinical management of patients. The third issue is MS data interpretation. During the preparation process, samples are prone to contamination by blood components or background tissue proteins, which will generate a long list of protein ‘hits’ [4]. Therefore, accurate quantitative analysis of all of the relative proteins in a protein profile is of great importance for correct subtyping. To date, all LMD/MS studies have used a ‘spectrum count’ to obtain quantitative information among different amyloid proteins; however, considering the fact that larger proteins can yield more observed peptides, this method may lead to misdiagnoses [11].

**Fig 1:**
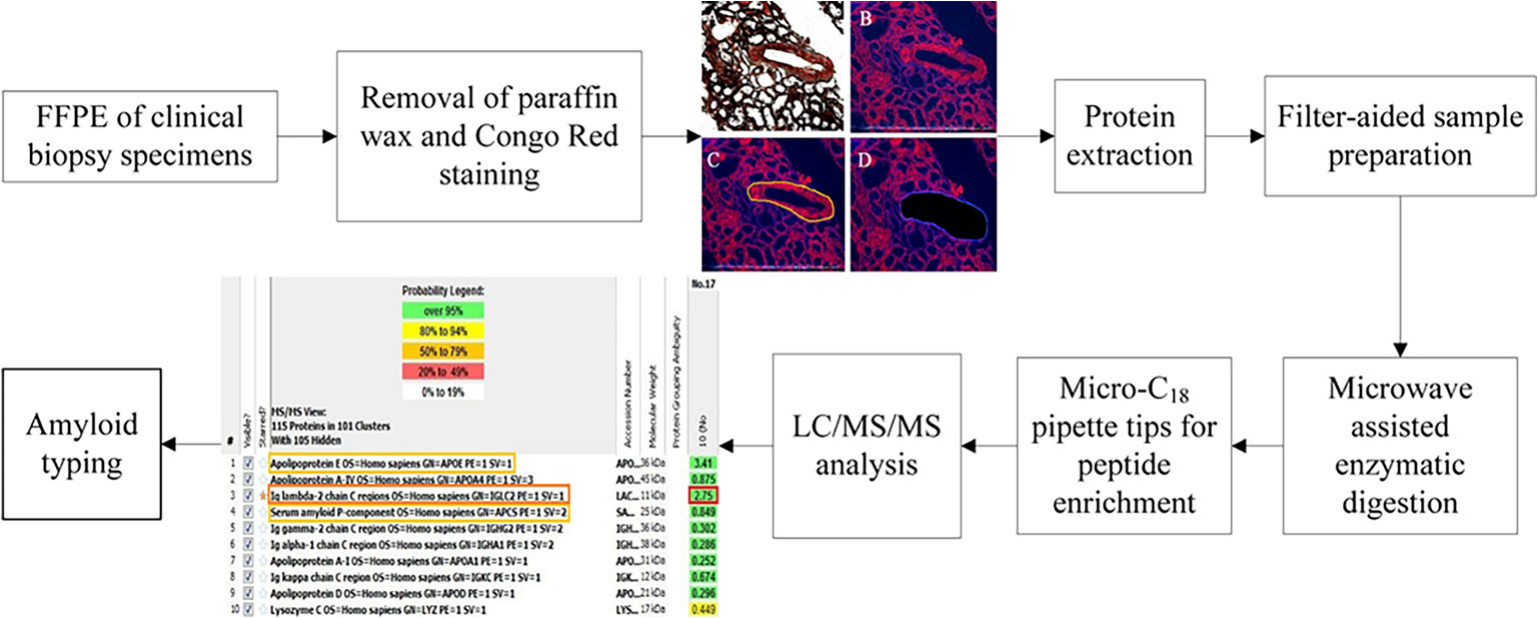
Workflow of the microwave-assisted filter-aided sample preparation (maFASP) method.

In this study, we developed a microwave-assisted filter-aided sample preparation (maFASP) method to analyze LMD samples. In this approach, a 10-kDa cutoff ultrafiltration unit was used to remove detergents and other low-molecular-weight contaminants during sample preparation [12–14], and protein digestion could then be completed in several minutes using a microwave-assisted digestion protocol [15]. Digested peptides were further purified by Micro-C_18_ ZipTip solid-phase extraction. Following LC/MS/MS analysis, an exponentially modified protein abundance index (emPAI) [11], which quantitatively considered the theoretical number of peptides per protein, was used to evaluate the relative quantitative abundance of each protein (Fig 1). To better interpret the results of the MS analysis, two novel parameters, P- and D-scores, were developed to depict the abundance of amyloid proteins and to identify the causative protein among a list of ‘hits’. Using this method, we completed sample preparation within four hours and successfully diagnosed amyloid subtypes in various clinical FFPE specimens.

## Material and Methods

### Clinical Specimens

Twenty FFPE specimens from different tissues in patients who were diagnosed with systemic amyloidosis at Peking Union Medical College Hospital were retrospectively analyzed using LMD/MS to validate this modified approach. Two criteria were used to confirm a diagnosis of amyloidosis as follows: clinical evidence of unexplained organ enlargement or dysfunction and the existence of amyloid fibrils that yield an apple-green birefringence under polarized light after binding to Congo red. All patient specimens exhibited multiple organ and tissue involvement and showed affinity for Congo red in at least one tissue biopsy. Specimen resources for the training group included abdominal fat, tongue, salivary gland, kidney, heart, gastrointestinal tract and lung. The subtype of each case was determined according to current diagnostic standards, including investigations of plasma cell disorders, and immunohistochemistry or immunofluorescence for immunoglobulin κ and λ light chain, SAA and TTR (Table A in S1 File). The training group consisted of five AL-κ and fifteen AL-λ. Eighteen FFPE tissues that were Congo red-negative and did not exhibit clinical evidence of systemic amyloidosis were used as control samples (Table B in S1 File). To further assess the feasibility of this modified LMD/MS approach, twenty-four FFPE samples of systemic amyloidosis were analyzed for amyloid typing (validation group) (Table C in S1 File). The Institutional Review Board at Peking Union Medical College Hospital, Chinese Academy of Medical Sciences, approved this study, and written informed consent for the use of tissue samples was obtained from all of the patients.

### Specimen Preparation and Laser Microdissection

For each case, 10-μm-thick sections of each FFPE specimen were collected on polyethylene naphthalate (PEN) membrane slides designed specifically for microdissection. Then, paraffin was removed from each sample, and tissues were stained with Congo red. The Congo red-positive areas were identified under a fluorescence light microscope and were microdissected with LMD using a Leica LMD6500 system. For each case, 200,000–300,000 μm^2^ Congo red-positive tissues were placed into 0.5 ml microcentrifuge tubes containing 40 μl of 10 mM Tris/1 mM EDTA/0.2% Zwittergent 3–16 buffer (Sigma, St. Louis, MO, USA). For samples in the control group, similar areas of Congo red-negative tissues were microdissected and collected. Subsequently, tissues were heated at 100°C for 60 min with occasional vortexing followed by 25 min of 40 kHz sonication in a water bath [9]. Following centrifugation, the upper lysate was placed into 10-kDa cut-off ultrafiltration units and centrifuged [12]. Then, 25 mM ammonium bicarbonate was added three times to the units to remove detergents and other contaminants. We added 100 μl of 25 mM ammonium bicarbonate followed by vortexing to dissolve the protein mixture. Samples were subsequently reduced with 20 mM dithiothreitol in a 37°C water bath for 60 min and were alkylated with 50 mM iodoacetamide at room temperature in the dark for 45 min. After washing three times with 25 mM ammonium bicarbonate, the sample was dissolved using 50 μl of 25 mM ammonium bicarbonate, and trypsin was added at a 1:20 enzyme-to-substrate ratio. Subsequently, the sample was fixed to a float plate, placed in a beaker containing 1,000 ml of water and heated in a microwave oven at 850 W for two rounds of 1 min each round [15]. During the digestion process, the protein sample was completely submerged in water, and the water temperature should be maintained below 37°C. Then, the peptide mixture was centrifuged to the bottom of the ultrafiltration unit, and the membrane was washed with 50 μl of 25 mM ammonium bicarbonate. Prior to LC/MS/MS analysis, the peptide mixture was desalted using ZipTip Micro-C_18_ pipette tips (Millipore, Bedford, MA, USA). The time for each step is shown in Table 1.

**Table 1:** The time to complete each step of sample preparation.

Step in sample preparation	Time (min)
Heating at 100°C	60
Sonication in a water bath	25
Ultrafiltration (three times)	15
Reduction by DTT	60
Alkylation by IAA	45
Ultrafiltration (three times)	15
Microwave-assisted digestion protocol	2
Ultrafiltration (one time)	5
Desalting using a ZipTip	15
Total	242

### MS-based Proteomic Analysis

The digested peptides were analyzed using a self-packed RP C_18_ capillary LC column (75 μm×100 mm, 3μm). The eluted gradient was 5–30% buffer B1 (0.1% formic acid, 99.9% ACN; flow rate, 0.3 μl/min) for 20 min. A TripleTOF 5600 system (AB SCIEX, USA) was used to analyze each sample. The MS data were acquired in the high-sensitivity mode using the following parameters: 30 data-dependent MS/MS scans per full scan; full scans were acquired at a resolution of 40,000, and MS/MS scans were acquired at 20,000; rolling collision energy, charge state screening (including precursors with a +2 to +4 charge state) and dynamic exclusion (exclusion duration 15 s); the MS/MS scan range was 250–1800 m/z, and the scan time was 25 ms. A Mascot database search engine (Matrix Science, London; version 2.3.02) was used for protein identification against the UniProt human database (www.uniprot.com, 84910 entries). Trypsin cleavage specificity was set, with a maximum of two missed cleavages allowed. Carbamidomethylation (C) was set as a fixed modification. The searches were performed using a peptide and product ion tolerance of 0.05 Da. Scaffold (v 4.3.2) was used to further filter the database search results using the decoy database method. Protein identifications were accepted when at least two unique peptides were detected and the false discovery rate at the protein level was less than 1% based on decoy database searching. To estimate the relative protein abundance in the analyzed samples, we used emPAI as a quantitative parameter. A higher emPAI value was indicative of greater protein abundance [11, 16].

### Data Interpretation

For each case, the amyloid-associated proteins, such as κ, λ, SAA, TTR, immunoglobulin heavy chain and fibrinogen-α were screened from the protein lists [3]. The presence of serum amyloid protein P (SAP) was required for the diagnosis of amyloidosis [8]. Quantitative information on each protein was based on the emPAI value, and typing of amyloidosis was based on the greatest abundance of protein that corresponded to the specific type of amyloid [8].

To better interpret MS data for amyloid typing, two parameters, the P- and D-scores, were proposed. The P-score was developed to estimate the relative protein abundance of amyloid-associated components in each sample. The P-score was defined as

$$\text{P-score}\;\left(\%\right)=\frac{\Sigma \;({\text{emPAI}}_{\text{amyloid}})}{\Sigma \;({\text{emPAI}}_{\text{total}})}\times 100,$$

where Σ(emPAI_amyloid_) was the summation of emPAI values for all identified amyloid-associated proteins, including accompanying proteins, such as SAP, ApoE, and ApoAIV, and causative proteins, such as immunoglobulin light and heavy chains, TTR, SAA, fibrinogen-α, and gelsolin [2]. The Σ(emPAI_total_) was the summation of the emPAI values for all of the identified proteins in an MS profile. The P-scores in the training group (n = 20) and the control group (n = 18) were compared using the Mann–Whitney U test. For P-score values, the receiver operating characteristic (ROC) curve and the area under the curve (AUC) were assessed.

When more than one amyloid causative protein was identified by MS analysis, the D-score was used to evaluate the abundance difference between the first- and second-most abundant proteins. The D-score was defined as

$$\text{D-score}=\frac{{\text{emPAI}}_{1\text{st}}–{\text{emPAI}}_{2\text{nd}}}{{\text{emPAI}}_{1\text{st}}},$$

where emPAI_1st_ and emPAI_2nd_ represented the emPAI value of the proteins with the first- and second-greatest abundance, respectively. The D-scores of cases in the training and validation groups were calculated.

## Results

### Clinical Characteristics of Cases in the Training and Control Groups

To assess the diagnostic efficiency of our approach, a training group that contained twenty cases with conclusive subtype diagnoses was used (Table A in S1 File). Among them, sixteen cases were multiple myeloma, and corresponding investigations, including serum protein electrophoresis and immune fixation electrophoresis, confirmed the existence of serum monoclonal immunoglobulin. The other four cases were systemic amyloidosis with renal involvement, and an immunofluorescence test of renal tissue provided conclusive information for the subtype diagnoses. Tissue resources of FFPE samples included abdominal fat (six cases), tongue (four cases), kidney (four cases), salivary gland (two cases), heart (one case), intestine (one case), gingiva (one case) and lung (one case).

The control group included normal tissues and tissues of common diseases, such as cysts and tumors, and the eighteen samples came from eight different biopsy tissues (Table B in S1 File). Tissue resources for the control group were comparable to those of the training group; thus, the tissue backgrounds were similar between the two groups. All samples were negative for Congo red staining.

### Amyloid Typing Based on LMD/MS Analysis in the Training and Control Groups

LMD/MS analyses were performed following the procedure illustrated in Fig 1 (a case of AL-λ amyloidosis is presented in Fig A in S1 File). Using our method, the time for sample preparation could be shortened to approximately four hours (Table 1), and the whole process for amyloid typing could be finished within five hours, which significantly accelerated the experimental time.

**Fig 2:**
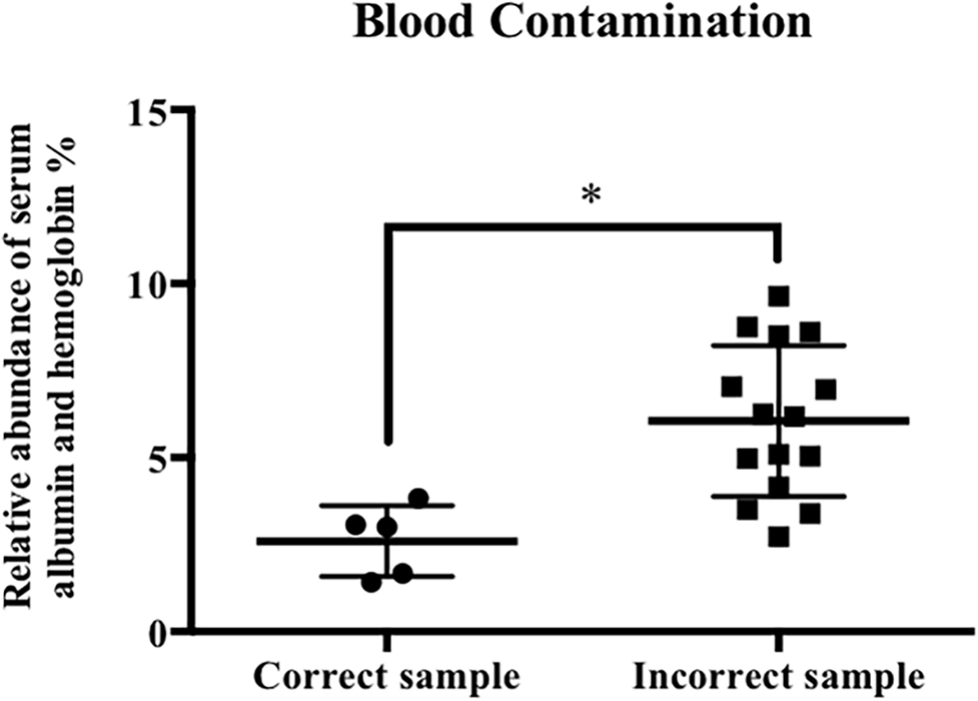
A comparison of the relative abundance of serum albumin and hemoglobin between correctly and incorrectly diagnosed cases in the training group based on the spectrum count method. The relative abundance of serum-related proteins of incorrectly diagnosed cases was significantly higher than that of correctly diagnosed cases; *P<0.05.

Initially, according to previous studies, the spectrum count was used to identify causative proteins in the MS profiles [7–10]. However, in the training group, based on the spectrum count method, 70% (14 of 20 cases) of AL amyloidosis were misdiagnosed as AH/AL+AH amyloidosis, and one case of AL-λ was misdiagnosed as fibrinogen-α amyloidosis (Table D in S1 File). Because numerous amyloid-associated proteins could be identified in the serum, once the samples were contaminated by blood, misdiagnoses were more likely to occur. Therefore, we compared the relative protein abundance of serum albumin and hemoglobin (×100%) between correctly and incorrectly diagnosed cases. The result indicated that the relative abundance of albumin and hemoglobin in incorrect cases was significantly higher than that in correct cases (Fig 2, Table D in S1 File), indicating that serum contamination was the primary reason for the identification of various amyloid-associated proteins. When more than one amyloid protein was identified, the spectrum count method tended to overestimate proteins with higher molecular weights because these proteins could produce more peptides [11]; thus, this method did not provide accurate quantitative information, resulting in misdiagnoses. Therefore, we employed the emPAI value as a surrogate.

In the training group, we successfully diagnosed five cases of AL-κ and fifteen cases of AL-λ according to the dominant amyloidogenic proteins. The results were completely coincident with previous clinical subtype diagnoses. Our approach diagnosed amyloid subtypes with 100% sensitivity. Detailed results of our current clinical diagnoses and LMD/MS analyses are summarized in Table 2. For the control group, a low level of peptides corresponding to the immunoglobulin light and heavy chains could be identified, while a minute amount of SAP, ApoE and ApoAIV components were detectable in twelve cases (Table 3).

**Table 2:** Subtype diagnosis based on the emPAI value in the training group.

Case	Tissue	MS profile (emPAI value)						P-score	D-score	Typing	Clinical
number	source	Ig κ C	Ig λ C	Ig α C	Ig γ C	TTR	Fibrinogen-α			result	diagnosis
1	Abdominal fat		2.3		1.1		0.31	15.30	0.52	AL-λ	AL-λ
2	Heart	1.17	3.17	0.286	1.48	1.16	0.225	21.56	0.53	AL-λ	AL-λ
3	Tongue	2.63			0.092		0.184	22.54	0.93	AL-κ	AL-κ
4	Intestine	0.674	2.75	0.667	0.259		0.0699	25.30	0.75	AL-λ	AL-λ
5	Abdominal fat	2.63	9.82	4.83	1.4	0.471	0.225	13.18	0.51	AL-λ	AL-λ
6	Abdominal fat	1.17	1.88	0.522	0.549		0.0699	20.99	0.38	AL-λ	AL-λ
7	Gingiva	3.69	1.21	0.522	1.28		0.184	10.69	0.65	AL-κ	AL-κ
8	Abdominal fat	0.674	1.21	0.183	0.33	0.213	0.107	7.28	0.44	AL-λ	AL-λ
9	Tongue	1.17	5.37	0.8	1.05	0.213	0.145	18.66	0.78	AL-λ	AL-λ
10	Tongue		1.21		0.4	0.471	0.107	24.29	0.61	AL-λ	AL-λ
11	Kidney	0.674	2.75	0.286	0.302			12.23	0.75	AL-λ	AL-λ
12	Kidney	1.17	3.89	0.183	0.306			22.24	0.70	AL-λ	AL-λ
13	Kidney		0.698		0.191		0.267	9.98	0.62	AL-λ	AL-λ
14	Kidney	0.294	0.698		0.0914	0.213		18.8	0.57	AL-λ	AL-λ
15	Salivary gland	1.8			0.419			8.24	0.77	AL-κ	AL-κ
16	Salivary gland	6.85		0.0876	0.69	0.783	0.31	27.97	0.89	AL-κ	AL-κ
17	Abdominal fat	1.17	0.698	0.522	0.419	0.213	0.145	11.18	0.40	AL-κ	AL-κ
18	Abdominal fat	0.674	2.75		0.845			22.13	0.69	AL-λ	AL-λ
19	Lung	1.8	2.75	0.655	0.845		0.0699	5.73	0.35	AL-λ	AL-λ
20	Tongue	2.63	3.89	0.286	0.933	1.16	0.145	11.18	0.32	AL-λ	AL-λ

C, constant region.

**Table 3:** MS profile of cases in the control group.

Case	MS profile (emPAI value)								P-score
number	SAP	ApoE	ApoAIV	Ig κ C	Ig λ C	Ig α C	Ig γ C	TTR	
1				0.674		0.8	0.579	0.471	2.39
2	1.36	0.299				0.286	0.259		3.45
3	0.446			1.8	1.21	0.522	0.845	1.16	4.21
4						0.183	0.191		3.49
5			0.322	0.674		0.183		0.471	0.78
6	0.12					0.0735	0.0509		1.34
7				0.674		0.183	0.419		1.25
8	0.446		0.15	1.8	0.69	0.655	0.69		4.24
9						0.183			1.23
10				1.17		0.286	1.01		3.06
11	0.279	0.191		1.8		0.348		0.783	3.27
12				1.8		0.294	0.191		3.84
13		0.418	0.631	5.07	0.761	0.409	0.724	1.16	7.11
14	1.67			1.17	0.698	0.522	1.23		6.72
15	0.635			1.17		0.183			1.09
16	1.09	0.191		1.8		0.286	0.464	0.783	2.55
17	1.09	1.85	1.48	2.63		0.399	1.4	1.16	8.18
18	0.446	0.299	0.15	1.8		0.522	1.16		3.27

C, constant region.

### Performance of the P- and D-scores in Amyloid Diagnosis and Subtyping

**Fig 3:**
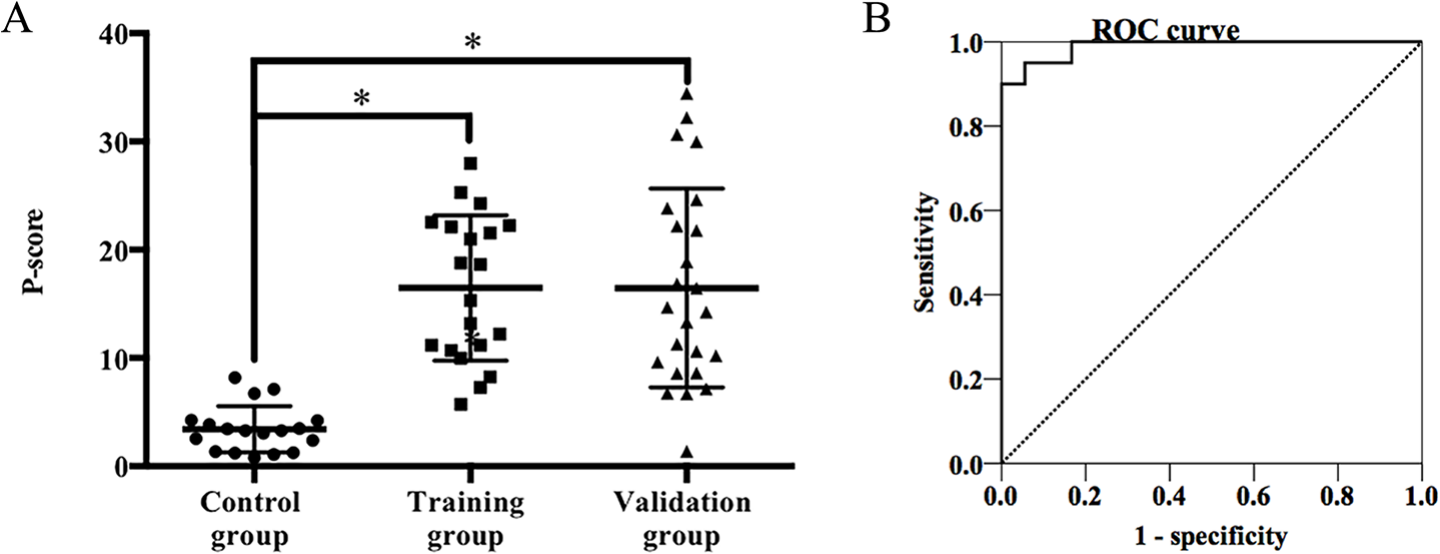
The establishment of the P-score value as a marker of amyloid deposits. A. A comparison of P-score values in the training, control and validation groups. P-score values of the training and validation groups were significantly higher than those of the control group; *P<0.05. B. An ROC curve for P-score values in the training and control groups (AUC = 0.971).

To better interpret the MS results, the P-score was proposed to estimate the existence of amyloid deposits. The hypothesis was that the proportion of amyloid-associated proteins in an amyloid deposit should be higher than that in a non-amyloid deposit. To test the performance of the P-score, we investigated this parameter in the training and control groups. The P-score in the training group ranged from 5.73 to 27.97 (average, 16.47); in the control group, it ranged from 0.78 to 8.18 (average, 3.42). Based on the Mann–Whitney U test, we found that the P-scores were significantly different in the amyloid versus the non-amyloid specimens (P<0.0001) (Fig 3A). Therefore, the P-score might serve as a surrogate marker to identify the existence of amyloid deposits. Based on the ROC curve (Fig 3B), when the P-score was 5.0, we could obtain a maximum sum of sensitivity (100%) and specificity (83.3%) for an amyloid diagnosis.

During sample preparation, a tissue might be contaminated by serum; thus, different causative proteins could be identified by MS analysis. To define the most abundant protein, we developed the D-score as a reference parameter. Once a diagnosis of amyloidosis was established, the D-score would contribute to successful typing when more than one target protein was identified. In the training group, the D-score ranged from 0.32 to 0.93. For all twenty cases in the training group, subtypes that were diagnosed based on amyloid causative proteins with the highest emPAI value were perfectly identical to the clinical diagnostic standards, so we adopted 0.30 as the threshold for the D-score. When the D-score was higher than 0.30, we detected an abundance difference between the dominant and secondary proteins that was associated with amyloidosis, which was sufficiently significant for subtype diagnoses. When the D-score was lower than this threshold, the possibility of serum contamination should be considered, and informative evidence from a clinical accessory test could be quite helpful in such a situation.

### Amyloid Subtype Diagnosis in the Validation Group

In the validation group, all cases had clinical evidence of organ involvement, and tissue biopsy showed positivity for Congo red staining. Because an increasing number of studies have highlighted the importance of measuring serum immunoglobulin free light chain (FLC) levels in the diagnosis and treatment monitoring of plasma cell proliferative disorders [17], seven of the twenty-four cases underwent an assay for FLC. Three cases (case 1, 2 and 6) had previously undergone immunohistochemistry tests; however, they all showed equivocal or inconclusive information. The other twenty-one cases did not undergo this test (Table C in S1 File). According to current diagnostic standards, none of these samples could be accurately subtyped. For six cases that presented positive results in the FLC assay (Table C in S1 File), there was clinical suspicion of AL amyloidosis. Sample resources for MS analysis included abdominal fat (five cases), gingiva (five cases), heart (three cases), liver (three cases), tongue (two cases), kidney (one case), pleura (one case), intestine (one case), salivary gland (one case), skin (one case) and laryngeal mass (one case).

The P- and D-scores were applied to the validation group to assist the subtype diagnosis. Based on our observations, the P-score was significantly higher in the validation group than in the control group (P<0.0001), whereas there was no significant difference between the validation and training groups (P = 0.814) (Fig 3A). Using a cut-off value, the P-scores of twenty-three (95.8%) cases were higher than 5.0, which indicated the existence of amyloid deposits and confirmed the results of Congo red staining. The P-score of case 11 was lower than 5.0 (P-score = 1.39). Although abundant immunoglobulin λ chain peptides had been identified by MS analysis, which was identical to the serum monoclonal component, a great abundance of peptides belonging to keratin were also detected in the protein profile. Considering the sample source of gingiva, contamination from background protein might be a reason for the lower proportion of amyloid proteins. Thus, further analysis is necessary to prove the diagnosis.

Among the twenty-three cases that had been verified with amyloid deposits, one case (case number 3) failed in amyloid typing as a result of abundant immunoglobulin κ and λ peptides that both existed in the protein profile with D-score lower than 0.30 (Table 4). Because the difference was not significant and we had no convincing clinical evidence for amyloid typing, the case failed in subtype diagnosis. According to the MS analysis, abundant serum albumin and hemoglobin were both identified; therefore plasma contamination was considered as a possible explanation for this failure.

**Table 4:** MS typing results of patients in the validation group.

Case	Tissue	MS profile (emPAI value)							P-score	D-score	Subtype
number	source	Ig κ C	Ig λ C	Ig α C	Ig γ C	TTR	SAA	Fibrinogen-α			diagnosis
1	Liver	1.8	1.88	0.655	1.4		4.4		32.2	0.57	AA
2	Pleura	1.17	0.303	0.655	0.509			0.107	9.63	0.44	AL-κ
3	Laryngeal mass	2.63	3.31	1.49	0.69	1.62		0.0699	23.85	0.21	Failure
4	Liver	2.63			0.419	0.213			16.44	0.84	AL-κ
5	Abdominal fat	1.17	0.303	0.399	0.617	2.18		0.604	14.24	0.46	ATTR
6	Liver	3.69		0.399	0.549			0.107	14.68	0.85	AL-κ
7	Gingiva	1.17		0.522	0.49			0.267	6.74	0.55	AL-κ
8	Salivary gland	2.63		0.183	0.81			0.145	10.61	0.69	AL-κ
9	Abdominal fat	0.674			0.56	5.88		0.145	24.63	0.89	ATTR
10	Heart	0.294		0.286	0.947				8.59	0.69	AH
11	Gingiva		1.21						1.39	1.00	Failure
12	Heart	1.8		0.522	0.549	25.5		0.0343	29.97	0.93	ATTR
13	Abdominal fat	1.17		0.191					10.21	0.84	AL-κ
14	Heart	0.674	1.21		0.166	0.213			13.32	0.44	AL-λ
15	Abdominal fat			0.183	0.361		2.14		18.89	0.83	AA
16	Tongue		1.21	0.183	0.361				21.79	0.70	AL-λ
17	Skin	3.69	1.21	0.655	1.2			0.184	22.19	0.67	AL-κ
18	Intestine	1.8		0.522	0.192	0.471			7.15	0.71	AL-κ
19	Abdominal fat	2.63	0.761	0.8	0.783			0.184	11.28	0.70	AL-κ
20	Gingiva	1.8	2.75	0.399	0.947				6.67	0.35	AL-λ
21	Gingiva	1.17		0.655	0.645	0.783		0.107	8.61	0.33	AL-κ
22	Gingiva	0.674	1.19	0.399	0.699				34.46	0.41	AL-λ
23	Tongue	1.17		0.183	0.361	4.67			16.84	0.75	ATTR
24	Kidney	1.17		0.399		0.471			30.64	0.60	AL-κ
1^*^	Gingiva	0.674		0.522	0.549	0.471			3.68	0.18	Non-amyloid
2^*^	Gingiva	0.674	1.21	0.183	0.419				23.17	0.44	AL-λ
3^*^	Tongue	0.674			0.699	1.16			4.4	0.40	Non-amyloid
4^*^	Kidney	0.674		0.399	0.69	2.18			4.2	0.68	Non-amyloid

Case 3 failed in diagnosis because the D-score was lower than 0.3.

Case 11 failed in subtyping because the P-score was lower than 5.0.

*Cases 1–4 with superscripts were Congo red–negative tissues that were adjacent to the Congo red-positive region of cases 21–24.

C, constant region.

In conclusion, amyloid subtypes of twenty-two samples were successfully identified in the validation group, including eleven cases of AL-κ, four cases of AL-λ, four cases of ATTR, two cases of AA and one case of AH. The diagnostic success ratio through an LMD/MS approach for this group was 91.7% (22/24 cases). For cases that had undergone immunoglobulin FLC assays, the subtypes diagnosed by MS were coincident with the serum evidence. Detailed information about the MS profile for each case in the validation group is shown in Table 4.

Finally, we also analyzed four Congo red-negative specimens that were adjacent to the region of amyloid deposits. Those samples were obtained from patients diagnosed as amyloidosis, with case numbers 21 to 24 in the validation group (Table 4). In all four cases, we detected SAP, and one surpassed the cut-off value for the P-score (P-score = 23.17). The protein profile of this case was consistent with AL-λ and was identical to the amyloid subtype diagnosed by the Congo red-positive sample. Although contamination may account for the positive result, this observation also indicated that MS analysis may provide a more sensitive diagnostic approach than Congo red staining. Additional studies are necessary to confirm the value of this method for amyloid diagnosis of Congo red-negative samples.

## Discussion

### Workflow Improvement

To improve the efficiency of detection, we successfully simplified and increased the speed of several steps in the typing process. First, we employed a filter-aided sample preparation (FASP) method prior to protein digestion [12]. The FASP method used a filter membrane with a selected molecular mass to retain high-molecular-weight substances, such as target proteins, and to remove low-molecular-weight substances, such as the detergents used for universal solubilization. Prior to the MS analysis step, peptide enrichment and a final cleanup were performed using pipette-tip microcolumns. The combination of FASP and this small-scale peptide enrichment protocol contributed to obtaining purified peptides, avoided interference from detergents in the MS analysis and extended the protein sequence coverage [13]. Recently, a FASP method was successfully applied in the proteomic characterization of FFPE tissues [14]. In that study, laser-captured microdissection followed by an FASP workflow and stage pipette-tip processing resulted in optimal protein identification. The study demonstrated that FASP was applicable in the proteomic analysis of complex FFPE samples. However, with the application of an ultrafiltration unit with a selected molecular mass, low-molecular-weight proteins would be lost during repeated centrifugations. Because the technique of laser microdissection could only provide a minute amount of tissue sample, whether amyloid deposits could be reliably processed and retain essential proteins for subtype diagnosis through the FASP method and LC/MS/MS analysis was still unknown. In this study, we used a filter unit with a relative molecular mass cut-off of 10-kDa as the FASP reactor together with C_18_ pipette-tip desalting microcolumns. According to our results, target proteins recognized as causative agents of amyloidosis, such as immunoglobulin light chains, SAA and TTR, were successfully identified, and their molecular weights were higher than 10 kDa. However, it was possible that certain small fragments of amyloid causative proteins were filtered out during sample processing, which may increase quantitative deviation. Therefore, ultrafiltration units with lower molecular cut-offs, such as 5000 Da or 1000 Da, may be more suitable for amyloid analysis.

Furthermore, we applied a microwave-assisted enzymatic digestion protocol, which is a straightforward method with established feasibility [15, 18], rather than the conventional tryptic digestion. Because the LMD/MS approach has demonstrated considerable potential as a diagnostic procedure, sample preparation is the most time-consuming step, as conventional tryptic digestion requires more than 16 hours [19], limiting the throughput of sample analyses. In our previous study, we reported that a microwave-assisted digestion strategy could enable highly efficient in-solution proteolysis of simple and complex protein samples in one minute; moreover, the digestion efficiency and LC/MS/MS results remained equivalent to standard overnight methods [15]. Yu et al. introduced a similar sample preparation approach using FASP and microwave-assisted on-filter enzymatic digestion for membrane proteome analysis [20]. The integrated workflow was shown to be ultrafast and efficient, indicating that microwave-assisted digestion and FASP can be used for complex sample preparation and are suitable for concurrent MS analysis [20]. In this study, the combination of FASP and microwave-assisted digestion was used for minute amounts of proteins from microdissected FFPE samples. The results indicated that maFASP successfully identified the protein components of minute samples through LC/MS/MS, verifying the robustness of this strategy, which may be applied for routine LMD sample preparation. Moreover, using maFASP reduced the sample preparation time to approximately four hours. However, laser microdissection represents an additional time-consuming step during sample processing. According to a previous study, LMD required 1–1.5 hour per slide even in experienced hands [21]. In this study, the average LMD time for each slide was appropriately one hour. A reduction of LMD time is challenging because highly accurate LMD is critically important for the entire procedure. However, our success in reducing the sample digestion time, which has previously required an additional one to two days, is helpful to improve the diagnostic efficiency.

Moreover, to establish diagnostic accuracy, emPAI was used for MS data interpretation. In most previous studies, the spectrum count was used as an indicator of protein abundance. However, serum contamination may result in diagnostic failure because larger proteins would generate more observed peptides [11]. When several amyloid-associated proteins were present, the abundance of larger proteins may be overestimated, as observed in the training group. EmPAI, which was first proposed by Ishihama and colleges in 2005 [11], considers the theoretical number of peptides per protein and thus can be used to more accurately estimate the protein abundance [11, 16].

To better interpret MS data, two parameters based on emPAI values, the P- and D-scores, were proposed. When the P-score was higher than the cutoff value, causative proteins from amyloid deposits could be accurately identified from the protein list, even in the presence of contaminating serum proteins. However, when the P-score was lower than the cutoff value, it was difficult to distinguish between causative proteins from amyloid deposits and amyloid-associated proteins from serum contamination; thus, the result was not appropriate for subtype diagnosis. Therefore, the P-score may indicate the quality of sample preparation and the reliability of interpretation of the results. Besides, we observed that one of four Congo red-negative samples adjacent to amyloid deposits could be successfully subtyped, which was consistent with the result from the Congo red-positive area. Although this conclusion requires additional validation, we speculate that the P-score may serve as a more sensitive method for the identification of amyloid deposits for Congo red-negative samples. Moreover, Vrana et al. have successfully used fresh abdominal subcutaneous fat tissues for amyloid subtyping [22]; for these tissues, the P-score may serve as a suitable parameter to indicate the quality of sample preparation. Additional studies are necessary to confirm this speculation. Subsequently, the D-score was helpful to identify the causative protein with the highest abundance. Using a combination of P- and D-scores, all twenty cases (100%) in the training group could be successfully subtyped. In the validation group, the success ratio of subtype diagnosis was 91.6%.

Compared with the study by Vrana et al. [7], our result was lower than the reported success ratio of 98%. In fact, amyloid deposits typically contain not only the major fibril constituents but also variable amounts of amyloid-associated proteins [4]. As previously mentioned, the most important issue in this study was serum contamination, which further complicated the interpretation of the results by generating complex protein lists. Therefore, the P- and D-scores were used to overcome this interference in amyloid subtyping. The introduction of scoring arithmetic guaranteed a correct diagnosis and clear discrimination between amyloidogenic proteins. However, a reduction in the diagnostic ratio was predictable. When the scoring arithmetic failed to identify the amyloid subtype, careful repeated processing of samples from the same tissue or other involved organs was recommended.

### Amyloid-associated Proteins in Amyloid Deposits

In our study, apart from causative proteins, such as the κ or λ light chain, SAP was identified as a coexisting protein in all twenty cases in the training group, while other proteins known to be integrated components in amyloid fibrils, such as apolipoprotein E, apolipoprotein A-I and A-IV, were also detected in most cases. It was noteworthy that in all twenty cases, low levels of immunoglobulin heavy chains were detected in protein profiles regardless of the amyloid subtypes. In fifteen cases, amyloid-associated proteins, such as transthyretin, fibrinogen-α and gelsolin, were also identified. Additionally, in AL cases, small amounts of immunoglobulin κ or λ light chains could be found in AL-λ or AL-κ samples, respectively, indicating that the two types of immunoglobulin light chains may coexist in laser-captured fibril deposits, although there was always significant variation in protein abundance. Similar observations were noted in the validation group. Immunoglobulin heavy chains were detectable in most cases, regardless of the subtypes for a case, while minute amounts of κ and/or λ light chains could also be found in ATTR and AA amyloidosis (Table 4). Similar observations have been reported in previous studies. In some cases, Vrana and colleges found that a small amount of κ light chain was present in addition to dominant amyloid proteins [7]. In another study focusing on renal amyloidosis, Sethi et al. reported that immunoglobulin heavy chains were present in most cases, whereas κ and/or λ light chains were also noted in AA, fibrinogen-αand Lect2 amyloidosis [8].

To explain the findings mentioned above, several reasons are possible. First, during the processing of an FFPE specimen, associated proteins, such as immunoglobulin molecules, may be introduced through blood contamination. In our study, various amyloid-related proteins were found to coexist, even in the samples of the control group, indicating that serum contamination represents a major problem for amyloid typing. Moreover, technique developments in the last several years have notably improved the performance of MS; thus, low levels of sample components can be detected. In this situation, MS will be able to identify minute amounts of contaminants. Second, substantial numbers of complete immunoglobulin molecules containing both light and heavy chains may be ‘trapped’ within amyloid fibrils that form from other causative proteins [23]. Finally, similar to non-fibrillar monoclonal immunoglobulin tissue deposition, substantial numbers of complete immunoglobulin molecules could be deposited along with amyloid fibrils.

### MS Performance in Amyloid Diagnosis

To date, the Congo red stain has been the gold diagnostic standard for amyloidosis. When bound to β-sheet-rich amyloid fibrils, Congo red molecules adopt a specific orientation. This spatial ordering gives the stain characteristics of birefringence and strong fluorescence [24]. Thus, it now serves as a specific diagnostic test in clinical processes.

Recently, Vrana et al. found that Congo red staining may yield false-negative results for amyloidosis, and MS analysis served as a useful alternative [25]. SAP is known to be a universal constituent of amyloid deposits [26], and components such as ApoE and ApoAIV also coexist with fibrils [7, 8]. Therefore, Vrana et al. proposed that the presence of a peptide representing at least two of the following three proteins, ApoE, SAP and/or ApoAIV, could be used as a more sensitive method than Congo red staining for the diagnosis of amyloidosis. Armed with this method, these authors successfully diagnosed amyloidosis with a sensitivity of 90% (330/366 cases) in Congo red-positive cases and 65% (13/20 cases) in cases that were negative for Congo red staining [25]. However, these authors also found one case (1/32 cases, 3.1%) without evidence of amyloidosis that could be misdiagnosed as amyloidosis using MS analysis, which they hypothesized was attributed to serum contamination [25]. Applying this 2/3 proteome signature to the training and control groups in our study, the diagnostic sensitivity and specificity were 75% and 61.1%, respectively. Because the 2/3 proteome signature method was based on the study of fresh tissue and serum contamination was generally observed in our study, therefore, this method may not be suitable for our results.

Considering all these findings, MS analysis can easily present false-positive results because of its high sensitivity and the possibility for serum contamination during sample processing, whereas Congo red stain is more specific to amyloidosis, but a false-negative result will impair diagnostic efficiency. In this study, we proposed two parameters (P- and D-scores) based on quantitative information regarding amyloid-related proteins. With the assistance of these two parameters, the diagnostic sensitivity and specificity of our approach were 100% and 83.3%, respectively; if combined with Congo red staining, these values would have both reached 100%. The analyses of Congo red-negative tissue (Table 4) indicated that our method may be more sensitive for amyloid diagnosis; however, future studies are necessary to confirm this conjecture to exclude sample contamination.

In conclusion, MS analysis performs well in amyloid diagnosis, and it is a preferable complement for existing methods. When serum contamination is excluded, the spectrum count method enables the accurate diagnosis and subtyping of amyloidosis, as previously reported. However, if contamination by serum proteins is observed, our study provides a practical solution. Once Congo red staining confirms the existence of an amyloid deposit, the detection of amyloid proteins with high P-score values can further validate the accurate acquisition of amyloid deposits. Subsequent subtype diagnosis is straightforward utilizing the D-score. When Congo red staining is equivocal or negative, but a suspicion of amyloidosis is strong, a P-score value greater than 5.0 provides a clue for repeated Congo red staining or a subsequent biopsy procedure of fat tissue or other involved organs.

### Future Work

Although we have provided a fast approach for amyloid typing, several important aspects could be improved in future studies. (1) The sample size analyzed in this study is relatively small, and a larger study with more samples derived from different tissue sources is necessary to demonstrate the efficiency of this method and to optimize related parameters. (2) Although we found that the emPAI value is superior to the spectrum count as an indicator of protein abundance, it remains a semi-quantitative scale and is unable to provide an accurate estimation of protein abundance. Notably, multiple reaction monitoring (MRM)-based MS analysis is a more sensitive and precise technique for the quantitative verification of target proteins in FFPE specimens [27, 28]. This technique could potentially be employed in this proteomic approach as a powerful new tool. However, one important issue should be noted prior to the application of MRM to maFASP samples. The peptide miscleavage rates using microwave-assisted digestion in a previous study (approximately 18%) and in our study (approximately 21%; detailed results are provided in Table E in S1 File) were higher than in overnight digests (14%) [20], indicating that less peptides could be selected as proteotypic peptides for MRM analysis. This aspect should be considered when selecting a proper sample preparation method for an MRM study. (3) The UniProt database was used in this study for protein identification, which included isoforms from one gene; however, proteins that contain point mutations and truncated proteins were not considered. Thus, a supplementary database including these proteins may be helpful to identify more peptides and provide more accurate quantitative results. (4) Notably, fresh tissues have been used for amyloid typing without an LMD process [22]; whether our method and parameters can be used in this protocol will require further investigation.

## Conclusion

In this study, we developed a maFASP method for amyloid typing using an LMD/MS approach with FFPE samples. Using this method, we could complete the sample preparation in four hours. According to the parameters (P- and D-scores) based on the emPAI value, we successfully subtyped 100% (20/20 cases) of the amyloidosis cases in the training group and 91.7% (22/24 cases) of the cases in the validation group. Moreover, our method provided a potentially alternative approach for amyloid diagnosis and typing for samples that presented false-negative results by Congo red staining.

Apart from AL, AA and ATTR, rare subtypes such as AH can also be identified accurately. For tissue sources beyond commonly involved organs, the results of this study extend the applications of LMD/MS to FFPE specimens derived from soft tissues, such as subcutaneous abdominal fat, tongue, gingiva, salivary gland and skin, which are easy to acquire and widely applicable. All of our results indicate that this method is reproducible and streamlined and demonstrate the potential to be widely clinically applied, particularly when serum contamination impairs the diagnostic efficiency of LMD/MS.

## Supporting Information

S1 File Supporting information of the maFASP method. LMD/MS analysis of a case of AL-λ amyloidosis (Figure A). Clinical features of patients in the training group (Table A). Clinical features of control cases (Table B). Clinical features and subtype diagnoses of patients in the validation group (Table C). A comparison of the spectrum counts and emPAI values for subtype diagnosis in the training group (Table D). Miscleavage rate of each sample from the three groups (Table E).
(DOCX)

S2 File Protein list of each sample from the three groups. (XLSX)

S3 File Peptide list of each sample from the three groups. (XLSX)

S4 File Protein list with emPAI values for each sample from the three groups. (XLSX)
