# Supplementary material for: The Successful Diagnosis and Typing of Systemic Amyloidosis Using A Microwave-Assisted Filter-Aided Fast Sample Preparation Method and LC/MS/MS Analysis
Source: PLoS One. 2015 May 18;10(5):e0127180. doi: 10.1371/journal.pone.0127180 (PMC4436214; doi:10.1371/journal.pone.0127180)
Supplement: S1 File — LMD/MS analysis of a case of AL-λ amyloidosis (Figure A). Clinical features of patients in the training group (Table A). Clinical features of control cases (Table B). Clinical features and subtype diagnoses of patients in the validation group (Table C). A comparison of the spectrum counts and emPAI values for subtype diagnosis in the training group (Table D). Miscleavage rate of each sample from the three groups (Table E). (DOCX) [file pone.0127180.s001.docx]

**Figure A.** LMD/MS analysis of a case of AL-λ amyloidosis. A. Renal FFPE specimen with interstitial amyloid deposition. (a) Thickened vascular wall was confirmed to be Congo red positive under light microscope. (b) Congophilic area showed bright red fluorescence under fluorescent light source. (c) Circled the selected area for micro-dissection in Leica LMD6500 system (Wetzler, Germany). (d) Area of amyloid deposition was micro-dissected and collected in 0.5ml microcentrifuge tube cap for following procedures. B. The list of proteins identified by mass spectrometry from micro-dissected fragments. Results were displayed by the use of Scaffold proteome software. Parameter of emPAI value was used as indicator of protein abundance. Diagnosis of amyloidosis was confirmed by the presence of SAP and ApoE components (indicated in orange text). According to the abundance of proteins represented in the sample, peptides belonging to immunoglobulin lambda chain constant regions were the most abundant (indicated in red text), so this case was typed AL-λ by MS analysis.


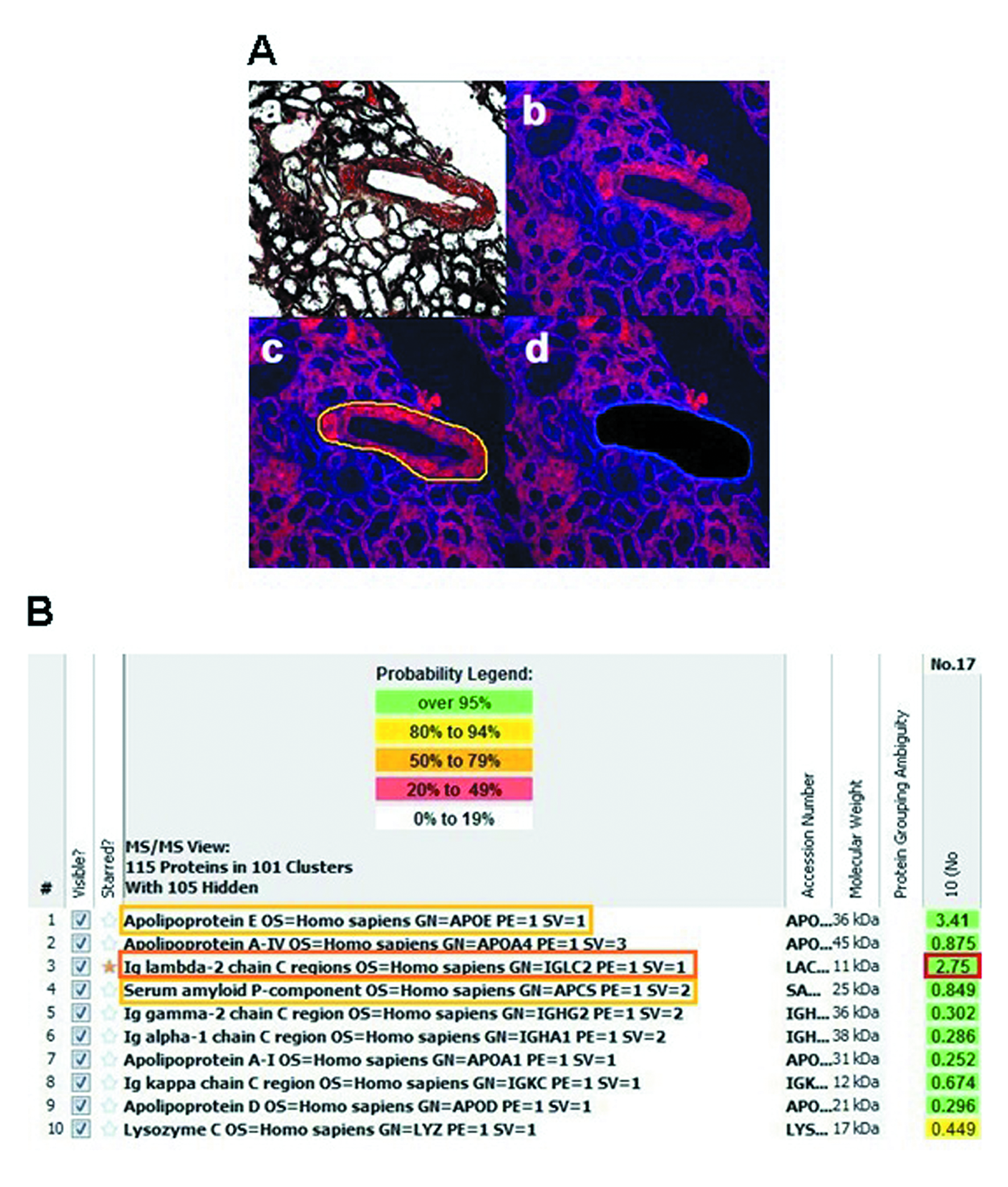


**Table A.** Clinical features of patients in the training group.

| Case | Age(y), sex | Amyloid organ involvement | Serum | Clinical | Amyloid |
| --- | --- | --- | --- | --- | --- |
| number |  |  | MC | evidence | subtype |
| 1 | 52, M | Heart, kidney, tongue, fat tissue | IgG λ | Along with MM | AL-λ |
| 2 | 55, M | Heart | IgG λ | Along with MM | AL-λ |
| 3 | 61, M | Heart, kidney, PNS, joint, tongue | κ | Along with MM | AL-κ |
| 4 | 63, M | Heart, GI, lymph node | IgA λ | Along with MM | AL-λ |
| 5 | 46, F | Kidney, fat tissue, lymph node | IgA λ | Along with MM | AL-λ |
| 6 | 65, F | Heart, tongue, gingiva, fat tissue | IgA λ | Along with MM | AL-λ |
| 7 | 47, F | Heart, gingiva | IgG κ | Along with MM | AL-κ |
| 8 | 61, M | Heart, kidney, liver, GI, skin, fat tissue | IgA λ | Along with MM | AL-λ |
| 9 | 51, M | Heart, PNS, lung, skin, tongue, gingiva | IgG λ | Along with MM | AL-λ |
| 10 | 60, M | Heart, tongue | IgG λ | Along with MM | AL-λ |
| 11 | 37, F | Kidney, salivary gland | IgM κ | Along with MM | AL-κ |
| 12 | 54, F | Heart, GI, joint, salivary gland | κ | Along with MM | AL-κ |
| 13 | 53, M | Heart, gingiva, fat tissue | IgG κ | Along with MM | AL-κ |
| 14 | 42, M | Heart, fat tissue | λ | Along with MM | AL-λ |
| 15 | 76, F | Lung | IgG λ | Along with MM | AL-λ |
| 16 | 62, M | Heart, tongue | λ | Along with MM | AL-λ |
| 17 | NA | Kidney | NA | IFR: AL-λ | AL-λ |
| 18 | NA | Kidney | NA | IFR: AL-λ | AL-λ |
| 19 | NA | Kidney | NA | IFR: AL-λ | AL-λ |
| 20 | NA | Kidney | NA | IFR: AL-λ | AL-λ |
| Case 17 to 20 were renal amyloidosis with robust immunofluorescence results, but some objective reasons, we failed to acquire detailed clinical information of those four cases.  Y, year; MC, monoclonal component; M, male; MM, case 1 to 14 were diagnosed as multiple myeloma according to gold diagnostic standard; PNS, peripheral nervous system; GI, gastrointestinal tract; F, female; NA, not available; IFR, immunofluorescence. | | | | | |

**Table B.** Clinical features of control cases.

| Case number | Tissue source | CR | Histology/Disease |
| --- | --- | --- | --- |
| 1 | Tongue | - | Glossal epidermoid cyst |
| 2 | Lung | - | Lung cancer |
| 3 | Stomach | - | Obesity |
| 4 | Fat tissue | - | Abdominal cicatrix |
| 5 | Kidney | - | Clear cell carcinoma of kidney |
| 6 | Heart | - | Normal heart muscle |
| 7 | Heart | - | Normal heart muscle |
| 8 | Gingiva | - | Gingival spindle cell carcinoma |
| 9 | Salivary gland | - | Labial mucous cyst |
| 10 | Salivary gland | - | Pleomorphic adenoma |
| 11 | Kidney | - | Clear cell carcinoma of kidney |
| 12 | Salivary gland | - | Pleomorphic adenoma |
| 13 | Kidney | - | Urothelial carcinoma |
| 14 | Lung | - | Interstitial lung disease |
| 15 | Stomach | - | Normal stomach tissue |
| 16 | Kidney | - | Renal cyst |
| 17 | Gingiva | - | Gingivitis |
| 18 | Heart | - | Normal heart muscle |
| CR, Congo red staining. | | | |

**Table C.** Clinical features and subtype diagnoses of patients in the validation group.

| Case | Age(y), sex | Amyloid organ involvement | Serum | κλ ratio | MS |
| --- | --- | --- | --- | --- | --- |
| number |  |  | MC | (0.26-1.25) | diagnosis |
| 1 | 31, F | Kidney, liver, spleen, GI, fat tissue | Neg |  | AA |
| 2 | 73, M | Heart, lung, skin | IgG κ |  | AL-κ |
| 3 | 50, F | Laryngeal mass | NA |  | AL-λ |
| 4 | 55, F | Kidney, liver | Neg | 6.65 | AL-κ |
| 5 | 47, M | Heart, PNS, GI, gingiva, fat tissue | NA |  | ATTR |
| 6 | 48, M | Heart, liver | Neg |  | AL-κ |
| 7 | 41, F | Heart, kidney, liver, gingiva | Neg |  | AL-κ |
| 8 | 37, M | Heart, kidney, PNS, liver, salivary gland | Neg |  | AL-κ |
| 9 | 61, M | Heart, PNS, fat tissue | NA |  | ATTR |
| 10 | 35, M | Heart | NA |  | AH |
| 11 | 68, M | Heart, joint, gingiva | λ |  | AL-λ |
| 12 | 46, M | Heart, PNS, skin | Neg |  | ATTR |
| 13 | 50, F | Kidney, skin, gingiva, fat tissue | Neg | 4.17 | AL-κ |
| 14 | 48, M | Heart, kidney | Neg |  | AL-λ |
| 15 | 35, F | Heart, lung, joint, salivary gland, fat tissue | Neg |  | AA |
| 16 | 81, M | Heart, kidney, tongue | λ | 0.035 | AL-λ |
| 17 | 24, F | Heart, PNS, skin | Neg |  | AL-κ |
| 18 | 59, M | Heart, kidney, liver, GI | Neg | 32.48 | AL-κ |
| 19 | 64, M | Liver, GI, gingiva, fat tissue | IgM κ | 6.172 | AL-κ |
| 20 | 58, F | Liver, gingiva | λ |  | AL-λ |
| 21 | 46, M | Gingiva, fat tissue | Neg |  | AL-κ |
| 22 | 50, F | Heart, PNS, tongue, gingiva | λ | 0.021 | AL-λ |
| 23 | 20, M | Heart, PNS, tongue, gingiva, CNS | Neg | 0.917 | ATTR |
| 24 | 41, F | Kidney |  |  | AL-κ |
| κλ ratio derived from the result of serum free light chain assay.  Y, year; F, female; Neg, negative; M, male; NA, not available; GI, gastrointestinal tract; PNS, peripheral nervous system; CNS, central nervous system. | | | | | |

**Table D.** A comparison of the spectrum counts and emPAI values for subtype diagnosis in the training group.

| Case | Tissue | Parameter | MS profile | | | | | | Typing | Clinical | Relative abundance |
| --- | --- | --- | --- | --- | --- | --- | --- | --- | --- | --- | --- |
| number | source |  | Ig κ C | Ig λ C | Ig α C | Ig γ C | TTR | Fibrinogen-α | result | diagnosis | of serum proteins (%) |
| 1 | Abdominal | Spectrum |  | 7 |  | 24 |  | 8 | AH | AL-λ | 8.5 |
|  | fat | EmPAI |  | 2.3 |  | 1.1 |  | 0.31 | AL-λ |  |  |
| 2 | Heart | Spectrum | 6 | 22 | 3 | 39 | 5 | 6 | AH | AL-λ | 4.97 |
|  |  | EmPAI | 1.17 | 3.17 | 0.286 | 1.48 | 1.16 | 0.225 | AL-λ |  |  |
| 3 | Tongue | Spectrum | 9 |  |  | 2 |  | 5 | AL-κ | AL-κ | 1.67 |
|  |  | EmPAI | 2.63 |  |  | 0.092 |  | 0.184 | AL-κ |  |  |
| 4 | Intestine | Spectrum | 2 | 8 | 9 | 3 |  | 2 | AH | AL-λ | 2.73 |
|  |  | EmPAI | 0.674 | 2.75 | 0.667 | 0.259 |  | 0.0699 | AL-λ |  |  |
| 5 | Abdominal | Spectrum | 9 | 13 | 39 | 32 | 2 | 6 | AH | AL-λ | 6.18 |
|  | fat | EmPAI | 2.63 | 9.82 | 4.83 | 1.4 | 0.471 | 0.225 | AL-λ |  |  |
| 6 | Abdominal | Spectrum | 3 | 5 | 5 | 6 |  | 2 | AH | AL-λ | 5.04 |
|  | fat | EmPAI | 1.17 | 1.88 | 0.522 | 0.549 |  | 0.0699 | AL-λ |  |  |
| 7 | Gingiva | Spectrum | 10 | 3 | 7 | 33 |  | 5 | AH | AL-κ | 5.10 |
|  |  | EmPAI | 3.69 | 1.21 | 0.522 | 1.28 |  | 0.184 | AL-κ |  |  |
| 8 | Abdominal | Spectrum | 2 | 4 | 2 | 5 | 1 | 3 | AH | AL-λ | 4.16 |
|  | fat | EmPAI | 0.674 | 1.21 | 0.183 | 0.33 | 0.213 | 0.107 | AL-λ |  |  |
| 9 | Tongue | Spectrum | 4 | 11 | 9 | 26 | 1 | 5 | AH | AL-λ | 7.04 |
|  |  | EmPAI | 1.17 | 5.37 | 0.8 | 1.05 | 0.213 | 0.145 | AL-λ |  |  |
| 10 | Tongue | Spectrum |  | 6 |  | 12 | 2 | 3 | AH | AL-λ | 9.64 |
|  |  | EmPAI |  | 1.21 |  | 0.4 | 0.471 | 0.107 | AL-λ |  |  |
| 11 | Kidney | Spectrum | 2 | 8 | 3 | 3 |  |  | AL-λ | AL-λ | 3.83 |
|  |  | EmPAI | 0.674 | 2.75 | 0.286 | 0.302 |  |  | AL-λ |  |  |
| 12 | Kidney | Spectrum | 3 | 9 | 2 | 5 |  |  | AL-λ | AL-λ | 3.00 |
|  |  | EmPAI | 1.17 | 3.89 | 0.183 | 0.306 |  |  | AL-λ |  |  |
| 13 | Kidney | Spectrum |  | 5 |  | 2 |  | 7 | AFib | AL-λ | 3.51 |
|  |  | EmPAI |  | 0.698 |  | 0.191 |  | 0.267 | AL-λ |  |  |
| 14 | Kidney | Spectrum | 1 | 5 |  | 1 | 1 |  | AL-λ | AL-λ | 1.42 |
|  |  | EmPAI | 0.294 | 0.698 |  | 0.0914 | 0.213 |  | AL-λ |  |  |
| 15 | Salivary | Spectrum | 4 |  |  | 4 |  |  | AL+AH | AL-κ | 6.25 |
|  | gland | EmPAI | 1.8 |  |  | 0.419 |  |  | AL-κ |  |  |
| 16 | Salivary | Spectrum | 12 |  | 1 | 6 | 3 | 8 | AL-κ | AL-κ | 3.07 |
|  | gland | EmPAI | 6.85 |  | 0.0876 | 0.69 | 0.783 | 0.31 | AL-κ |  |  |
| 17 | Abdominal | Spectrum | 3 | 2 | 6 | 4 | 1 | 4 | AH | AL-κ | 8.61 |
|  | fat | EmPAI | 1.17 | 0.698 | 0.522 | 0.419 | 0.213 | 0.145 | AL-κ |  |  |
| 18 | Abdominal | Spectrum | 2 | 7 |  | 8 |  |  | AH | AL-λ | 6.96 |
|  | fat | EmPAI | 0.674 | 2.75 |  | 0.845 |  |  | AL-λ |  |  |
| 19 | Lung | Spectrum | 4 | 6 | 7 | 9 |  | 2 | AH | AL-λ | 3.39 |
|  |  | EmPAI | 1.8 | 2.75 | 0.655 | 0.845 |  | 0.0699 | AL-λ |  |  |
| 20 | Tongue | Spectrum | 5 | 10 | 3 | 13 | 5 | 4 | AH | AL-λ | 8.76 |
|  |  | EmPAI | 2.63 | 3.89 | 0.286 | 0.933 | 1.16 | 0.145 | AL-λ |  |  |
| MS profile showed amyloid associated proteins identified by MS analysis.  C, constant region. | | | | | | | | | | | |

**Table E.** Miscleavage rate of each sample from the three groups.

| Case number | Miscleavage rate (%) | Case number | Miscleavage rate (%) | Case  number | Miscleavage rate (%) | Case number | Miscleavage rate (%) |
| --- | --- | --- | --- | --- | --- | --- | --- |
| Training-1 | 14.62 | Control-1 | 24.32 | Validation-1 | 20.90 | Adjacent-1 | 19.39 |
| Training-2 | 19.97 | Control-2 | 20.51 | Validation-2 | 16.75 | Adjacent-2 | 23.01 |
| Training-3 | 21.76 | Control-3 | 29.72 | Validation-3 | 22.68 | Adjacent-3 | 27.32 |
| Training- 4 | 14.59 | Control-4 | 20.71 | Validation-4 | 22.95 | Adjacent-4 | 22.58 |
| Training-5 | 22.85 | Control-5 | 23.92 | Validation-5 | 19.26 |  |  |
| Training-6 | 22.30 | Control-6 | 27.20 | Validation-6 | 20.99 |  |  |
| Training-7 | 19.03 | Control-7 | 32.27 | Validation-7 | 16.78 |  |  |
| Training-8 | 18.53 | Control-8 | 22.18 | Validation-8 | 25.11 |  |  |
| Training-9 | 20.84 | Control-9 | 20.41 | Validation-9 | 19.60 |  |  |
| Training-10 | 27.59 | Control-10 | 22.47 | Validation-10 | 13.81 |  |  |
| Training-11 | 21.61 | Control-11 | 24.86 | Validation-11 | 25.30 |  |  |
| Training-12 | 19.64 | Control-12 | 22.67 | Validation-12 | 15.72 |  |  |
| Training-13 | 18.33 | Control-13 | 26.47 | Validation-13 | 22.51 |  |  |
| Training-14 | 18.69 | Control-14 | 23.07 | Validation-14 | 22.60 |  |  |
| Training-15 | 16.88 | Control-15 | 28.07 | Validation-15 | 17.59 |  |  |
| Training-16 | 19.45 | Control-16 | 34.91 | Validation-16 | 16.84 |  |  |
| Training-17 | 23.99 | Control-17 | 30.14 | Validation-17 | 18.84 |  |  |
| Training-18 | 19.29 | Control-18 | 20.93 | Validation-18 | 23.07 |  |  |
| Training-19 | 14.22 |  |  | Validation-19 | 21.90 |  |  |
| Training-20 | 21.18 |  |  | Validation-20 | 16.92 |  |  |
|  |  |  |  | Validation-21 | 20.20 |  |  |
|  |  |  |  | Validation-22 | 26.63 |  |  |
|  |  |  |  | Validation-23 | 26.63 |  |  |
|  |  |  |  | Validation-24 | 25.24 |  |  |
